# ProBDNF Collapses Neurite Outgrowth of Primary Neurons by Activating RhoA

**DOI:** 10.1371/journal.pone.0035883

**Published:** 2012-04-27

**Authors:** Ying Sun, Yoon Lim, Fang Li, Shen Liu, Jian-Jun Lu, Rainer Haberberger, Jin-Hua Zhong, Xin-Fu Zhou

**Affiliations:** 1 Department of Human Physiology and Centre for Neuroscience, Flinders University, Adelaide, Australia; 2 Department of Anatomy and Histology and Centre for Neuroscience, Flinders University, Adelaide, Australia; 3 Division of Health Science, Sansom Institute, School of Pharmacy and Medical Sciences, University of South Australia, Adelaide, Australia; Indiana University School of Medicine, United States of America

## Abstract

**Background:**

Neurons extend their dendrites and axons to build functional neural circuits, which are regulated by both positive and negative signals during development. Brain-derived neurotrophic factor (BDNF) is a positive regulator for neurite outgrowth and neuronal survival but the functions of its precursor (proBDNF) are less characterized.

**Methodology/Principal Findings:**

Here we show that proBDNF collapses neurite outgrowth in murine dorsal root ganglion (DRG) neurons and cortical neurons by activating RhoA via the p75 neurotrophin receptor (p75NTR). We demonstrated that the receptor proteins for proBDNF, p75NTR and sortilin, were highly expressed in cultured DRG or cortical neurons. ProBDNF caused a dramatic neurite collapse in a dose-dependent manner and this effect was about 500 fold more potent than myelin-associated glycoprotein. Neutralization of endogenous proBDNF by using antibodies enhanced neurite outgrowth *in vitro* and *in vivo,* but this effect was lost in p75NTR^−/−^ mice. The neurite outgrowth of cortical neurons from p75NTR deficient (p75NTR^−/−^) mice was insensitive to proBDNF. There was a time-dependent reduction of length and number of filopodia in response to proBDNF which was accompanied with a polarized RhoA activation in growth cones. Moreover, proBDNF treatment of cortical neurons resulted in a time-dependent activation of RhoA but not Cdc42 and the effect was absent in p75NTR^−/−^ neurons. Rho kinase (ROCK) and the collapsin response mediator protein-2 (CRMP-2) were also involved in the proBDNF action.

**Conclusions:**

proBDNF has an opposing role in neurite outgrowth to that of mature BDNF. Our observations suggest that proBDNF collapses neurites outgrowth and filopodial growth cones by activating RhoA through the p75NTR signaling pathway.

## Introduction

Neuronal polarization involving neurite outgrowth and axonal elongation is essential for building functional neural circuits during brain development [1], [2]. Both positive and negative signals regulate the neurite outgrowth and guide axons to their appropriate destinations. Mature neurotrophins (NTs) including nerve growth factor (NGF), brain-derived neurotrophic factor (BDNF) and NT-3, NT-4/5 are well characterized positive signals promoting neurite outgrowth, axonal extension, filopodial protrusion and synaptogenesis [3], [4].

Proneurotrophins are proteolytically cleaved to form biologically active mature molecules. Recent studies illustrate that the neurotrophin precursors, proNGF, proBDNF, and proNT3 trigger apoptosis of sympathetic and sensory neurons to antagonize the effects of mature neurotrophins [5], [6], [7], [8]. ProBDNF is found to be a negative regulator of synaptic plasticity and regulates long-term depression via p75NTR [9], [10]. In addition, it negatively regulates the migration of cerebellar granule cells during development and the infiltration of macrophages during spinal cord injury [11], [12]. ProBDNF has distinct functions on different populations of neurons, reducing the number of cholinergic fibers and hippocampal dendritic spines without affecting the survival of these neurons [10]. However, the proBDNF dependent regulation of neurite outgrowth and the underlying signaling are not known.

A number of factors and signal pathways have been identified to negatively regulate neurite outgrowth or repulse the growth cones to cause neurite collapse during development and after nerve injury in the central nervous system (CNS). These include the myelin associated factors Nogo, myelin-associated glycoprotein (MAG) and oligodendrocyte-myelin glycoprotein (OMgp) which activate Nogo receptors (NgR) and its coreceptor p75NTR in RhoA dependent manner [13], [14]. Additional neurite growth inhibitory factors such as semaphorin3A, ephrin-B3 or repulsive guidance molecule b repulse the regeneration of CNS neurons [15], [16], [17], [18]. Understanding of the functions of molecules which regulate neurite outgrowth not only sheds the light on the development of nervous system but also helps to identify potential therapeutic targets for the promotion of CNS regeneration.

We hypothesize that proBDNF plays opposite roles to those of mature BDNF in neuronal functions. As mature BDNF is a potent molecule promoting neurite outgrowth and is an essential chemoattractant for axonal extension, proBDNF may counteract and balance the effects of mature BDNF on neurite growth. In the present study, we have used primary sensory and cortical neurons to test the hypothesis and were able to demonstrate that exogenous and endogenous proBDNF collapse neurite outgrowth by activating the small GTPase RhoA and its downstream effector Rho kinase (ROCK) via p75NTR.

## Results

### ProBDNF Collapses Neurites in a Dose-dependent Manner on Cortical and DRG Neurons

**Figure 1:**
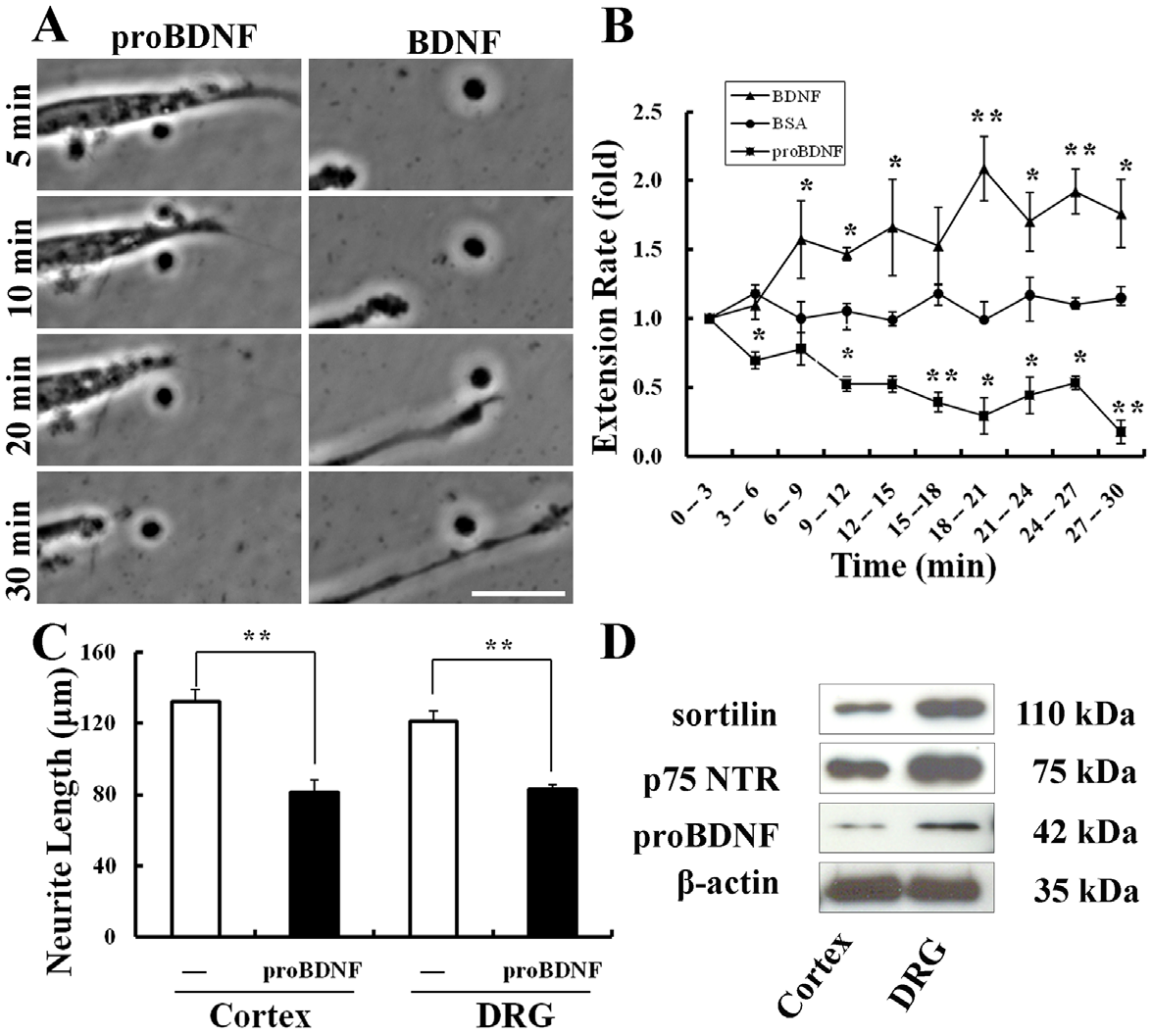
ProBDNF treatment decreases neurite length on DRG or cortical neurons. A, Time-lapse recordings show the collapsed neurite growth with proBDNF (left), and enhanced neurite growth with BDNF (right). Frames indicate 5, 10, 20, 30 min respectively. Scale bar, 20 µm. B, Treatment of DRG neurons with proBDNF decreased the rate of neurite extension within 3 min and remained decelerated through 30 min. *n* = 30 neurites/treatment. *p<0.05, **p<0.001, compared to BSA; Studen
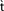
s *t* test. C, Treated cortical or DRG neurons with proBDNF caused similar collapse in neurite length. *n* = 83–85 neurons/treatment. *p<0.05, **p<0.001, significantly different from untreated neurons; Studen
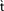
s *t* test. D, Expression of sortilin, p75NTR on the lysate of cultured cortical or DRG neurons processed for Western blot. Bands of 35 kDa of proBDNF, 75 kDa of p75NTR, and 110 kDa of sortilin were detected with their respective antibodies. ß-actin (42 kD) antibody was used as internal protein loading control. *n* = 3 independent experiments.

To demonstrate a role of proBDNF in neurite outgrowth, we first investigated its effects on DRG neurons. Live imaging clearly showed the collapse of neurites in response to proBDNF (30 ng/ml, Figure S1) and the enhanced neurite growth in response to mature BDNF (50 ng/ml, Figure S2, Fig. 1A). ProBDNF caused a 30±6% decrease in the neurite length after 6 min (*p*<0.05) which was maintained throughout the 30 min treatment (Fig. 1B). To investigate whether the proBDNF-dependent neurite collapse also occurs in CNS neurons, we examined its effect on cultured cortical neurons. The treatment with proBDNF induced in cortical neurons a decrease in neurite length similar to DRG neurons (Fig. 1C). To investigate if the effect could be mediated via endogenous release of proBDNF and subsequent interaction with its receptors, we determined the protein expression levels of proBDNF, p75NTR and sortilin in primary cultured cortical and DRG neurons [19]. ProBDNF was detected as a single band at 35 kD, p75NTR at 75 kD and sortilin at 110 kD. ß-actin with a single band at 42 kD was used as an internal loading control (Fig. 1D).

### Endogenous Probdnf Inhibits Neurite Growth Through p75NTR

**Figure 2:**
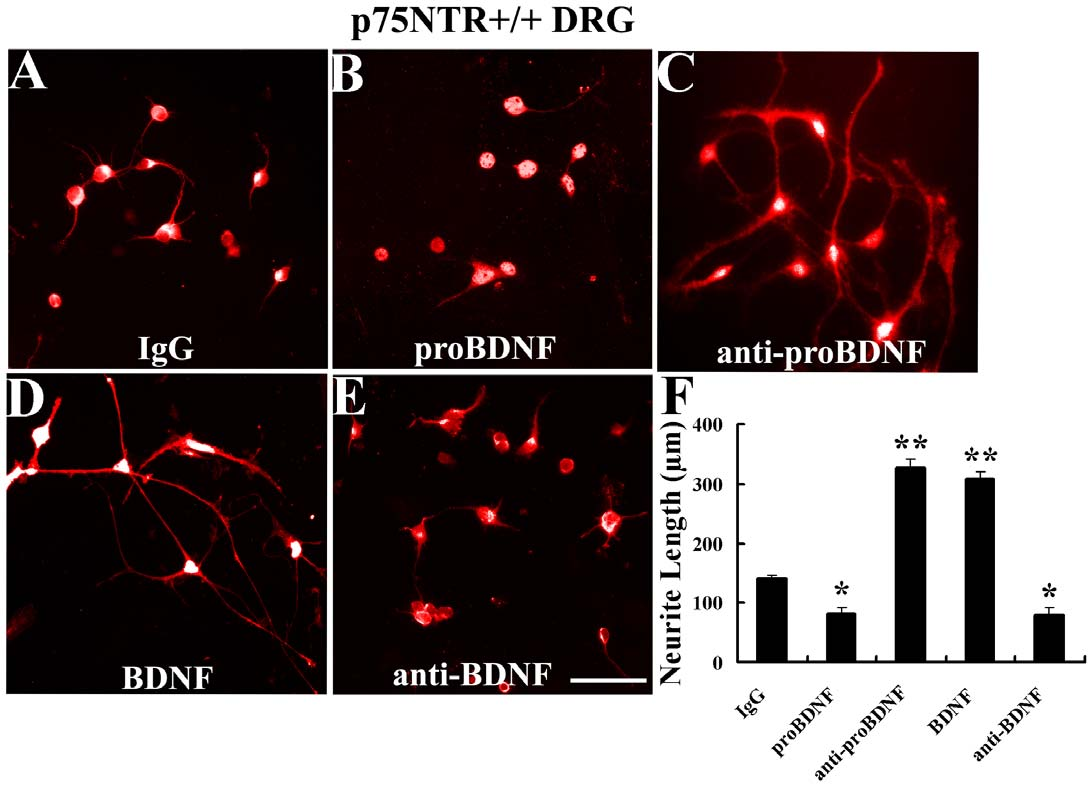
ProBDNF decreases neurite length on DRG neurons *in vitro*. Cultured DRG neurons were treated by different factors as shown, immunofluorescence stained by anti-MAP2 antibody. A, Normal sheep IgG. B, ProBDNF. C, Anti-proBDNF. D, BDNF. E, Anti-proBDNF. F, The neurite outgrowth assay were analyzed under the different culture conditions. Scale bar, 50 µm. *n* = 89∼92 neurons/treatment from three independent dishes. *p<0.05, **p<0.01, compared to IgG; one-way ANOVA with least-significant difference *post hoc* test.

In this assay, cultured primary adult DRG neurons were treated with different factors (Fig. 2A–E). The neurite length was significantly reduced in presence of proBDNF (82.7±9 µm) but increased in presence of anti-proBDNF antiserum (326.3±14 µm) or mature BDNF (307.8±12 µm) compared to the IgG control (139.4±8 µm, *p*<0.05, Fig. 2F). The results suggest that both exogenous and endogenous proBDNF inhibits neurite outgrowth on DRG neurons while mature BDNF enhances the neurite growth.

**Figure 3:**
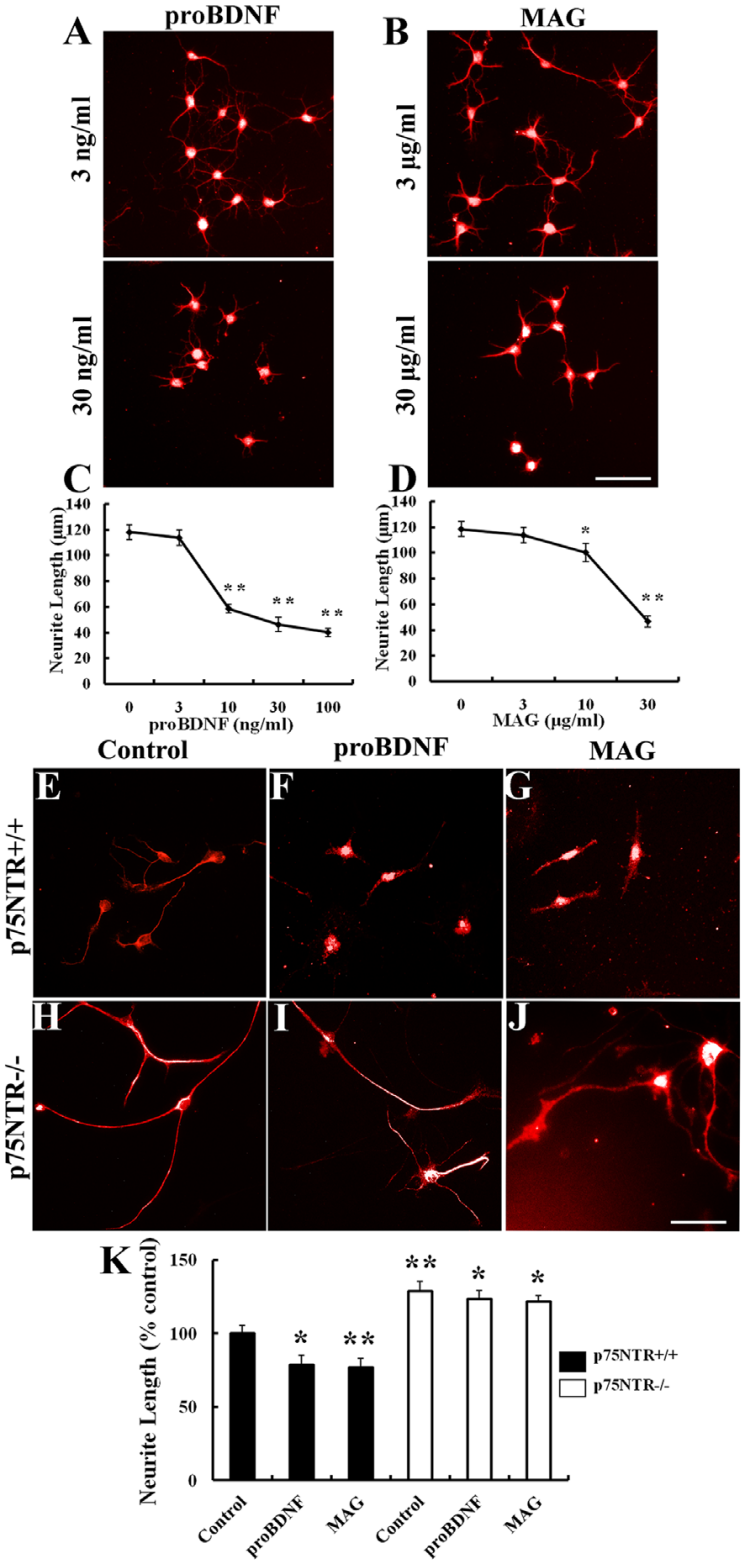
ProBDNF collapses neurite length *in vitro* through p75NTR. A, Cultured DRG neurons were treated by proBDNF (3 ng/ml, 30 ng/ml) or myelin-associated glycoprotein (MAG 3 µg/ml, 30 µg/ml), respectively, immunofluorescence stained by anti-MAP2 antibody. C, proBDNF decreased neurite length on DRG neurons in a dose-dependent manner. **p<0.01, compared to untreated neurons; Studen
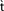
s *t* test. D, MAG decreased neurite length of DRG neurons in a dose-dependent manner. *p<0.05, **p<0.01, compared to untreated neurons; Student’s *t* test. E–G, Anti-MAP2 staining of p75NTR^+/+^ DRG neurons treated by control, proBDNF, MAG, respectively. H–J, Anti-MAP2 staining of p75NTR^−/−^ DRG neurons treated by control, proBDNF, MAG, respectively. K, ProBDNF or MAG treated p75NTR^+/+^ DRG neurites displayed significant length decrease compared with control group. However, proBDNF or MAG treated p75NTR^−/−^ DRG neurites had no effect compare with control group. Scale bar, 50 µm. *n* = 85∼90 neurons/treatment. *p<0.05, **p<0.01, compared to control; one-way ANOVA with least-significant difference *post hoc* test.

The application of proBDNF resulted in dose-dependent decrease in sensory neuron neurite length compared to untreated DRG neurons (Fig. 3A, C) with IC50 is about 10 ng/ml. The p75NTR is also the co-receptor for Nogo receptor which binds the inhibitory molecule MAG. This allowed us to directly compare the potency of proBDNF and MAG on the inhibition of the neurite outgrowth in primary cultured DRG neurons. The application of MAG (10, 30 µg/ml) resulted in a small but significant decrease in neurite length (100±7 µm, 46±4 µm) compared with the control group (118.4±6 µm, *p*<0.05), whereas a lower concentration of MAG (3 µg/ml) to DRG neurons had no effect (Fig. 3B, D). The concentration of 30 µg/ml MAG-Fc (MW: 120,000, 250 nM) has an equivalent effect of, 30 ng/ml proBDNF (dimer MW: 64,000∼70,000, 0.42∼0.46 nM). Thus proBDNF is 533∼583 fold more potent than MAG.

To further investigate whether the p75NTR signaling is involved in proBDNF effects we applied proBDNF to neurons dissected from p75NTR^−/−^ and p75NTR^+/+^ mice. The application of proBDNF (30 ng/ml) or MAG (30 µg/ml) decreased the neurite length by 21±7% and 23±6% in primary cultured p75NTR^+/+^ DRG neurons (*p*<0.01, Fig. 3E–G). However, the treatment of p75NTR^−/−^ DRG neurons with proBDNF or MAG had no effect on neurite outgrowth (*p*>0.05, Fig. 3H–K).

### RhoA Activity is Increased After ProBDNF Treatment but Lost in p75NTR^−/−^ mice

**Figure 4:**
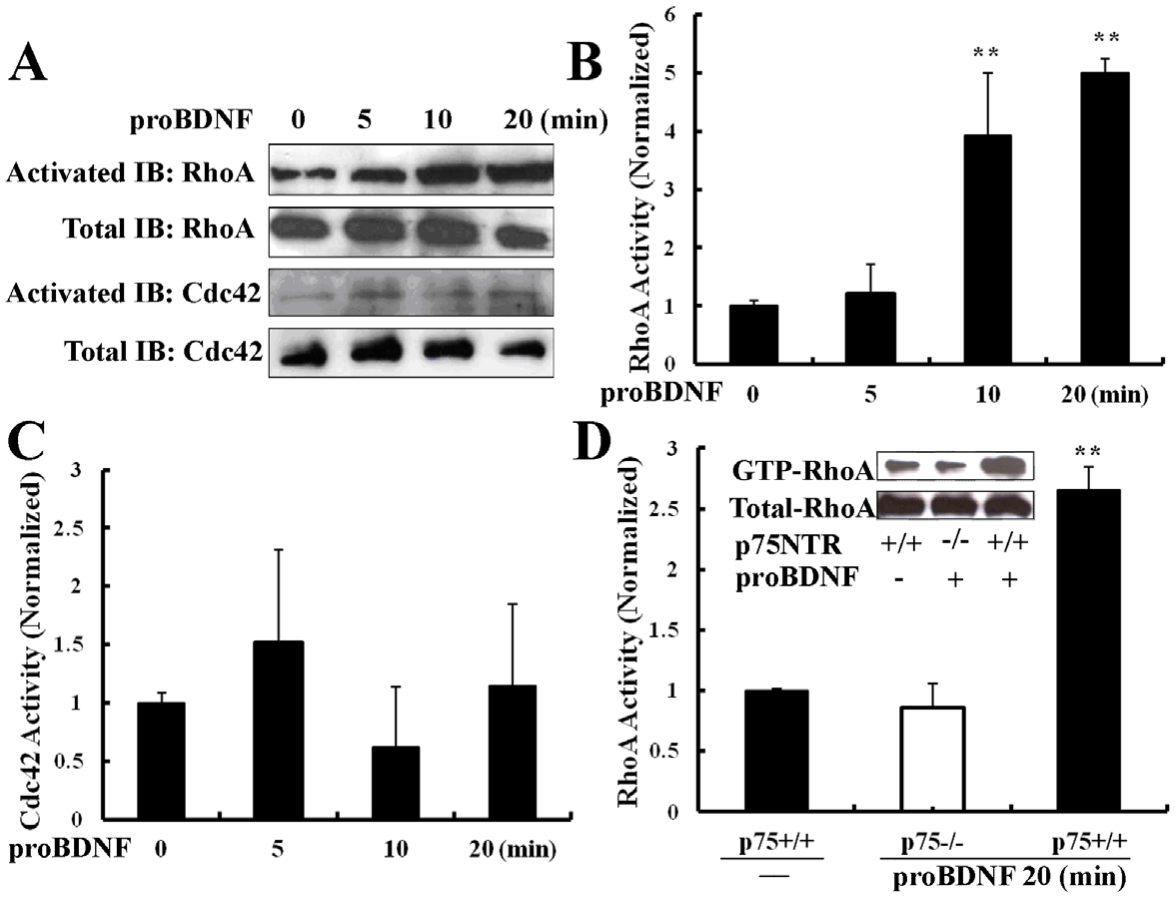
ProBDNF increased RhoA activity in cultured cortical neurons through p75NTR. A, Activated and total levels of RhoA or Cdc42 with proBDNF (0, 5, 10, 20 min) treatment which were processed for immunoblot (IB) assay. B, 10 and 20 min exposure to proBDNF caused significant production in activated RhoA normalized to total RhoA. **p<0.01, compared to untreated neurons; Studen
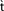
s *t* test. C, Exposure to proBDNF caused no differences in activated Cdc42 normalized to total Cdc42. D, RhoA activity increased on p75NTR^+/+^ cortical neurons after treated by proBDNF 20 min compared with p75NTR^+/+^ control neurons whereas p75NTR^−/−^ cortical neurons exhibited no difference. *n* = 3∼4 independent experiments. **p<0.01, compared to control level; Studen
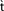
s *t* test.

It is known that MAG and Nogo cause neurite collapse by activating RhoA and this effect is mediated by a direct interaction of the Rho GDP dissociation inhibitor (Rho-GDI) with p75NTR [20]. Because proBDNF binds p75NTR with high affinity, we next tested the hypothesis that proBDNF collapses neurites by activating RhoA. After treatment with proBDNF for 10 and 20 min, RhoA activity increased 3.9±1.2 and 4.9±0.4 fold (Fig. 4A–B). Interestingly, no change in the level of activated Cdc42 was seen in response to proBDNF (Fig. 4C). Based on these observations we used the pull-down activity assay to compare RhoA activity in cortical neurons with or without proBDNF from p75NTR^+/+^ (*n* = 12) and p75NTR^−/−^ mice (*n* = 6). The results showed that, in contrast to cells from p75NTR^−/−^ mice, proBDNF increased RhoA activation in cells from p75NTR^+/+^ mice over untreated cells (2.6±0.2 fold, *p*<0.01, Fig. 4D).

### ProBDNF Activates RhoA and Induces the Collapse of Growth Cone Filopodia

**Figure 5:**
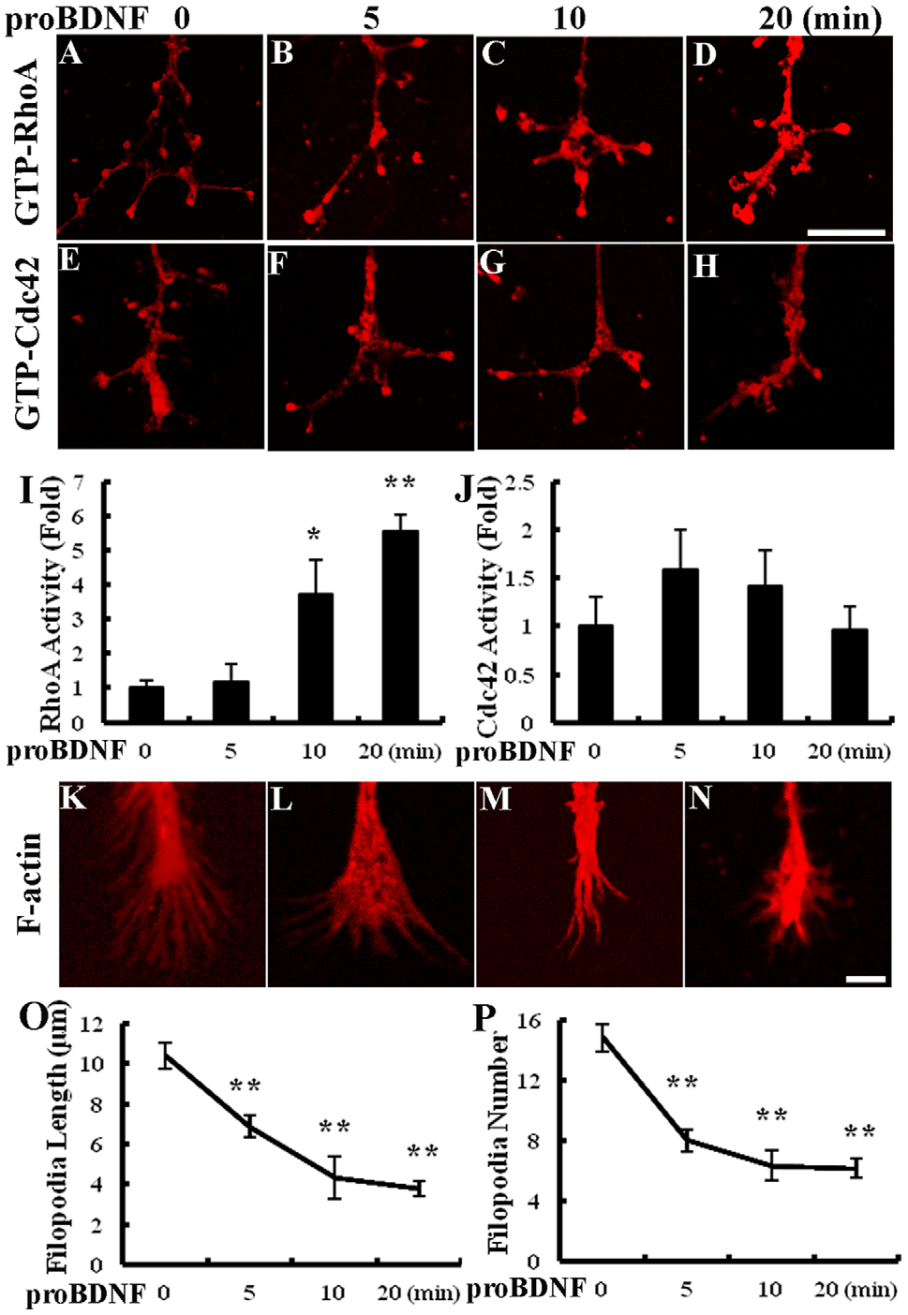
ProBDNF treatment increases RhoA activity and filopodial length on DRG growth cones. A–H, Immunoreactivity of RhoA or Cdc42 activity in DRG growth cones after proBDNF (0, 5, 10, 20 min) treatment. Scale bar, 10 µm. I, Quantification of RhoA activity. There were significant inductions after exposure to proBDNF 10, 20 min. *p<0.05, **p<0.01, compared to untreated neurons; Studen
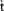
s *t* test. J, Quantification of Cdc42 activity. There was no change during the whole 20 min exposure of proBDNF. K–N, F-actin of DRG growth cones after proBDNF (0, 5, 10, 20 min) treatment. Scale bar, 5 µm. O, ProBDNF decreased filopodial length on DRG growth cones in a time-dependent manner. P, ProBDNF decreased filopodia number on DRG growth cones in a time-dependent manner. *n* = 35 neurites/treatment from at least three independent experiments. **p<0.01, compared to untreated neurons; Studen
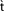
s *t* test.

We detected activated RhoA using specific GTP-RhoA antisera. As shown in Fig 5A–D, proBDNF treatment caused a time-dependent increase in GTP-RhoA immunoreactivity in growth cones. After treatment with proBDNF for 10 and 20 min, GTP-RhoA immunoreactivity increased 3.7±0.9 and 5.5±0.4 fold in growth cones (Fig. 5I) whereas the immunoreactivity for GTP-Cdc42 antibody was similar at the different time points (Fig. 5E–H, 5J).

We next determined whether proBDNF regulates filopodial length and number (Fig. 5K–N). Filopodia collapsed in response to proBDNF correlates with RhoA activation in growth cones. After 5, 10 and 20 min, proBDNF caused a significant retraction of filapodial length from 10.3±0.6 µm to 6.8±0.5 µm, 4.3±1.0 µm and 3.8±0.9 µm (*p*<0.01, Fig. 5O), and decreased the number of filapodia 8±0.7, 6.3±1.0, 6.2±0.6 (*p*<0.01, Fig. 5P) relative to untreated neurons (14.8±0.9).

### ProBDNF Induced Inhibition of Neurite Outgrowth Depends on the Activation of RhoA and ROCK

**Figure 6:**
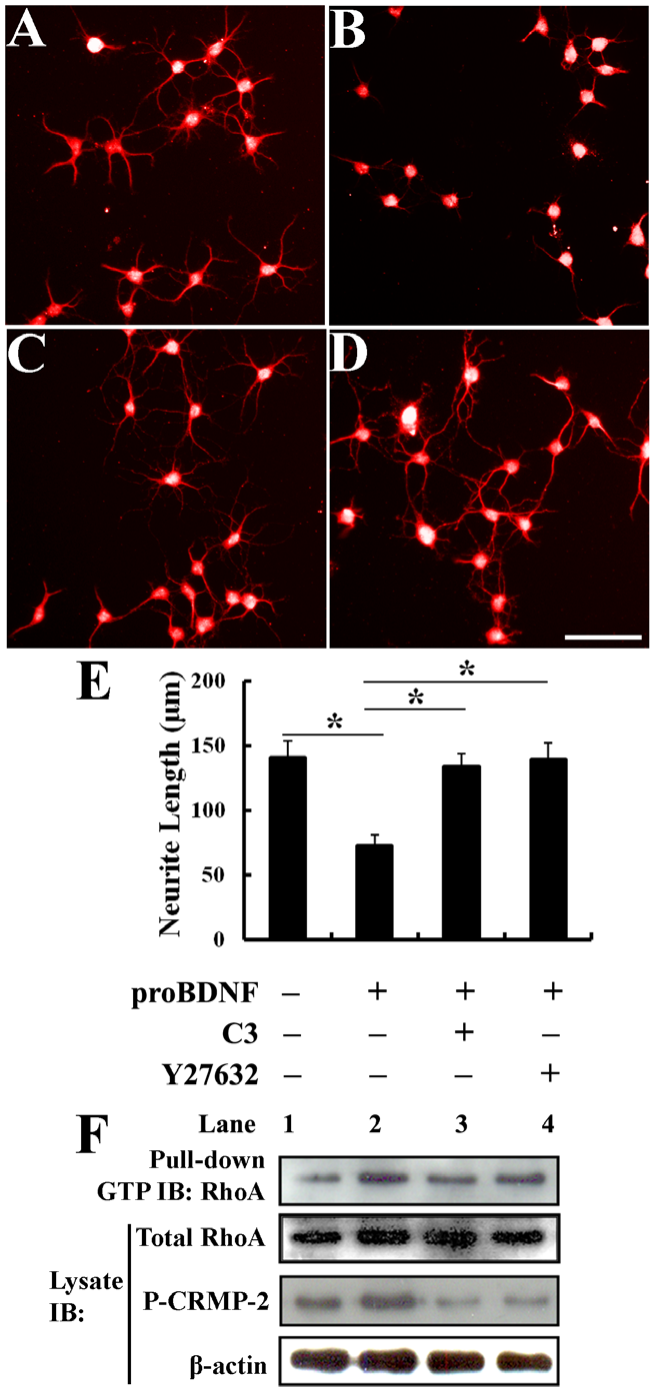
ProBDNF inhibits neurite growth through activation of RhoA and ROCK. Cultured DRG neurons were treated by different factors, followed by labelling of anti-MAP2 using immunocytochemistry. A, Treatment with normal sheep IgG. B, DRG neurons treated with proBDNF have shorter neurites. C, Pretreatment with C3-transferase (RhoA inhibitor) reverses proBDNF induced inhibition of neurite growth. D, Y27632 (ROCK inhibitor) completely reverses the proBDNF induced inhibition. Scale bar, 50 µm. E, Neurite outgrowth assay. ProBDNF significantly reduced the neurite length whereas pretreatment with C3 or Y27632 restored the reduction. *n* = 91∼95 neurons/treatment from three independent experiments. *p<0.01; Studen
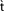
s *t* test. F, GTP-RhoA was measured by pull-down assay. Activated CRMP-2 and total RhoA and ß-actin are also shown by immunoblot (IB). Lane 1, normal sheep IgG control group. Lane 2, proBDNF-induced RhoA activation. Lane 3, The RhoA inhibitor C3 abolishes proBDNF-induced RhoA activation. Lane 4, ROCK inhibition Y27632 blocks proBDNF-induced RhoA activation. Phosphorylation of the collapsin response mediator protein-2 (P-CRMP-2) was assessed with phospho-specific antisera. ß-actin was used as internal protein loading control. *n* = 3 independent experiments.

Next, we asked whether RhoA and ROCK signaling is involved in proBDNF-mediated inhibition of neurite outgrowth. C3-transferase (a molecule that ADP ribosylates RhoA) and Y27632 (a well characterized ROCK inhibitor) were used to inactivate RhoA and inhibit ROCK, respectively. Functional studies using the neurite outgrowth assay showed that short-term incubation (20 min) of cultured DRG neurons with proBDNF significantly inhibited neurite outgrowth compared with control IgG (Fig. 6A, B). The extent of inhibition induced by pharmacological inhibitors of RhoA and ROCK were slightly different (Fig. 6C, D). C3-transferase reversed the inhibitory effects by 95%, whereas Y27632 abolished the inhibitory effect of proBDNF completely (Fig. 6E). GTP-bound RhoA or phosphorylation levels of the collapsin response mediator protein-2 (CRMP-2) were increased in response to proBDNF but their responses were abolished by preincubation of these cells with C3 or Y27632 for 30 min and subsequent stimulation with proBDNF for 20 min (Fig. 6F).

### ProBDNF Decreases the Nerve Innervations of Ventral Planta of the Hind Limb *in Vivo* Through p75NTR

**Figure 7:**
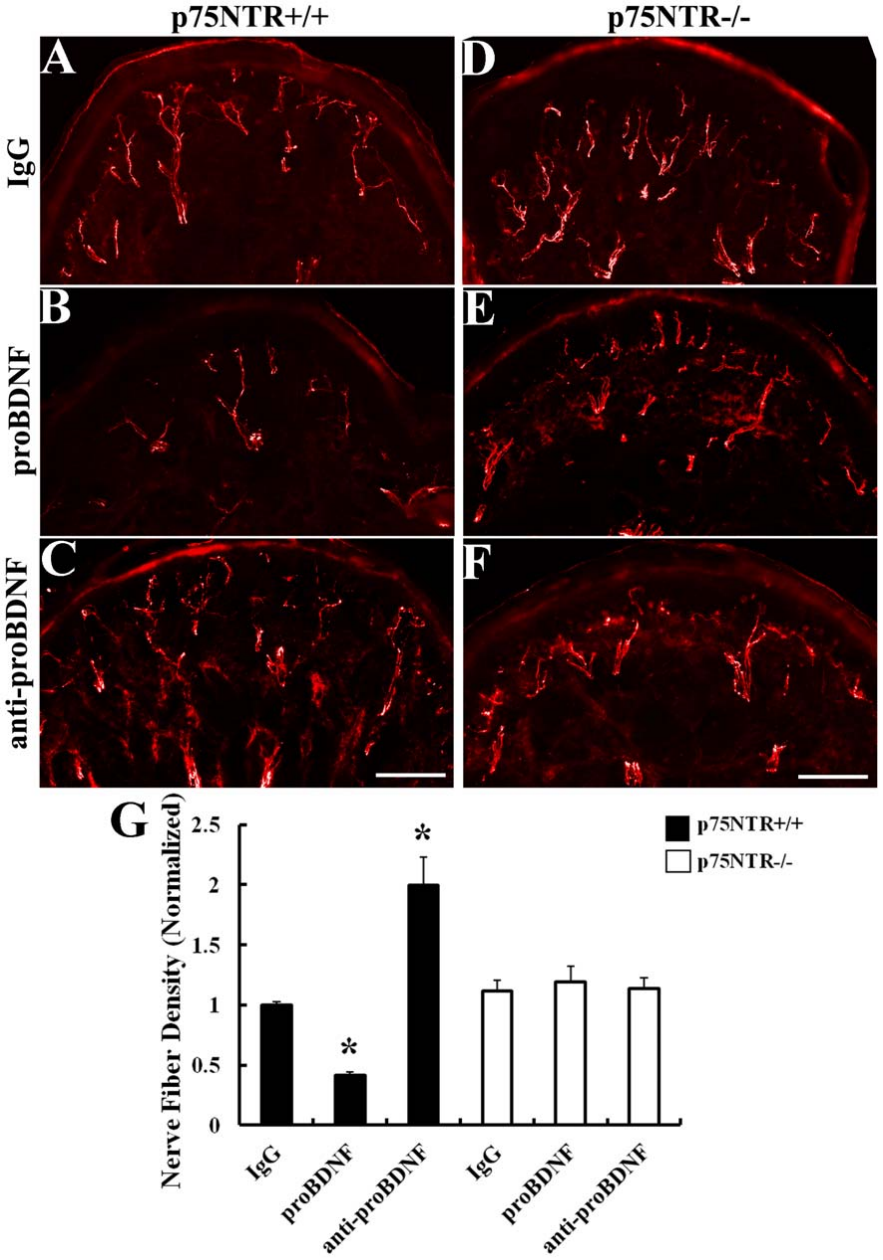
ProBDNF decreases the nerve innervation in ventral planta skin *in vivo* through p75NTR. A–C, Anti-neurofilament staining of footpad treated by normal sheep IgG (IgG), proBDNF, anti-proBDNF, respectively on p75NTR^+/+^ postnatal 5 days animals. D–F, Anti-neurofilament staining of footpad treated by IgG, proBDNF, anti-proBDNF, respectively on p75NTR^−/−^ postnatal 5 days animals. G, Quantification of nerve fiber density. The maximal threshold of control IgG fiber density (area occupied/total area of field of view) was defined as 1, for the treatments, density of the fibers was divided by the IgG fiber density in the same optical field. ProBDNF reduced the nerve fibers density while anti-proBDNF increased the nerve fibers density on p75NTR^+/+^ animals. However, proBDNF or anti-proBDNF had no effect in p75NTR^−/−^ mice. Scale bar, 100 µm. *n* = 40 sections/treatment from five independent animals. *p<0.01, compared to IgG; Studen
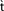
s *t* test.

Finally, we examined whether proBDNF had a similar effect on fiber growth *in vivo*. Postnatal day 5 (P5) p75NTR^+/+^ and p75NTR^−/−^ mice were injected with proBDNF, anti-proBDNF or IgG via footpad for 3 days. At P10, the density of total nerve fibers in the ventral planta was immunostained and quantified (Fig. 7A–F). Interestingly, in p75NTR^+/+^ pups, more nerve fibers could be seen in the anti-proBDNF group (2.0±0.2 fold, *p*<0.001) compared with the IgG group. In the proBDNF group, the fiber density was significantly decreased (0.4±0 fold, *p*<0.001), indicating the fibers retracted after proBDNF treatment. However, this effect was absent when p75NTR^−/−^ mice were treated with proBDNF (Fig. 7G). This suggests that endogenous proBDNF may play a negative role in the innervations of tissue *in vivo* through p75NTR.

## Discussion

The morphology of axons and dendrites of neurons depends on the dynamics of the cytoskeleton which is regulated by diffusing factors from the environment, extracellular matrix, cell surface receptors and intracellular signals [21]. BDNF and other mature neurotrophins are important factors regulating neurite outgrowth and neuronal differentiation during development. However, the physiological function of proneurotrophins such as proBDNF in neurite outgrowth remains unclear. Our hypothesis is that endogenously released neuronal proBDNF may oppose functions of mature BDNF [22], [23], [24]. ProBDNF is highly expressed in DRG neurons and cortical neurons, can change transport anterogradely and retrogradely [25], [26], [27], and acts as a negative regulator via the p75NTR for the migration of cerebellar granule cells [11]. In the present study using a live cell imaging methods and immunostaining, we show that proBDNF is a potent neurite collapsing factor. The effect of proBDNF on neurite growth of neonatal cortical neurons is the same as in adult sensory DRG neurons. Most importantly, we have found that the neutralization of proBDNF with antibodies which recognize the prodomain of proBDNF shows a very robust increase in neurite outgrowth both *in vitro* and *in vivo*. The effect of the neutralization antibody on neurite growth is at least comparable to that of mature BDNF. Despite of some controversy regarding proBDNF distribution and release [28], [29], proBDNF has been detected in a number of brain regions by Western blot and immunohistochemistry [27], [30]. These studies strongly indicate that endogenous proBDNF may have physiological and pathological functions on axonal growth and guidance.

Neurotrophins bind two kinds of receptors, tropomyosin receptor kinase (Trk) family and p75NTR, to mediate diverse functions during nerve development and in the adult brain. p75NTR is normally co-expressed with all Trks and modulates the Trks survival response of neurons. On the other hand, p75NTR is the coreceptor of NgR and the p75NTR/NgR complex mediates neurite outgrowth inhibition *in vitro* by MAG, OMgp, and Nogo [13], [14], [18]. Thus, during the development, neurite outgrowth depends on the balance mediated by p75NTR between positive signals stimulated by neurotrophins and negative signals from these myelin components in their environment [20], [31]. Simple deletion of p75NTR causes a retardation of axonal elongation in the periphery of developing mice [32] but significantly increases axonal growth in the central nervous system [33], indicating the importance of environmental factors in neurite growth regulated by p75NTR. In the present study we have identified that proBDNF is a potent neurite collapsing factor which activates RhoA via p75NTR to regulate neurite growth. As proBDNF and BDNF are anterogradely transported and are likely released concomitantly together in an activity dependent manner [22], [23], [24], proBDNF may play an antagonizing role in axonal elongation and dendritic arborization. Our *in vivo* data showed that blocking endogenous proBDNF dramatically increased the neurite outgrowth in the ventral planta, supporting the notion that proBDNF may be a physiological factor counteracting mature BDNF to maintain the balance of innervating territory. Recent studies by Miller’s group showed that p75NTR regulates dendritic, axonal pruning and axonal degeneration during development [18], [34]. Extracellular shedding of p75NTR can antagonize the inhibitory effect of myelin associated proteins on neurite outgrowth [35], indicating the soluble extracellular domain of p75NTR can block inhibitory factors. As p75NTR and sortilin are coreceptors for proBDNF [19], [36] and both are expressed in primary sensory neurons and primary cortical neurons, proBDNF may act as an autocrine or paracrine factor. In this study, we confirmed that the proBDNF, p75NTR and sortilin, are highly expressed on DRG and cortical neurons. Our studies show that altering the balance by application of anti-proBDNF antibodies to neutralize endogenous proBDNF can promote neurite outgrowth and axonal elongation, suggesting that proBDNF is normally released and functional. As no effect of anti-proBDNF is seen in DRG neurons of p75NTR^−/−^ mice, it is most likely that the full length proBDNF, rather than the prodomain fragment which does not bind the p75NTR, works via p75NTR to cause neurite collapse.

To further confirm the inhibitory effect of proBDNF on neurite growth, we used MAG as the positive control [37], [38] to compare their effects. Both dose-dependently inhibit neurite growth but proBDNF is 533∼583 fold more potent than MAG. As endogenous myelin-associated inhibitors are considered major impediments to regeneration after nerve injury, a myriad of strategies are applied to suppress these factors for promoting regeneration [39], [40], [41]. However the results are disappointing as the elimination of MAG, Nogo, OMgp or Semaphorin-mediated inhibition are not sufficient to promote extensive axon repair after spinal cord injury [41], [42]. These studies suggest that these inhibitors do not play a major role in CNS axon degeneration [43]. In the present study, we found that proBDNF is over 500 more potent than MAG in collapsing neurites, suggesting that proBDNF can be one of the potent inhibitors for nerve regeneration after CNS injury. In particular, when axons are injured, proBDNF is anterogradely transported to the injury sites then accumulates in the injured axonal bulb in the spinal cord [25]. It is likely that the accumulated proBDNF is released to collapse growth cones of CNS axons and prevent the regeneration of injured neurons. Thus the suppression of endogenous proBDNF and other proneurotrophins following CNS injury may promote regeneration of injured nerves.

Filopodia can detect environmental cues to transduce signals which then guide growth cone directions and dynamics [21], [44]. Growth cone turning was induced by the guidance cue, BDNF, and by the repellent factor, MAG [45]. In this study, we confirmed that proBDNF participates in growth-cone morphology and motility by reducing the length and number of filopodia. It is also likely that during development, proBDNF may function to repulse axons and balance the effect of mature BDNF to guide axons to their right destination. This is consistent with our previous work demonstrating that proBDNF repulses the migration of cerebellar granule neurons and their neurites, whereas mature BDNF or proBDNF antibodies attract them [11].

Growth cone protrusion and retraction of neurons is due to the dynamic assembly and disassembly of tubulin and actin cytoskeletal proteins. RhoA, Cdc42, Rac1 GTPases provide the necessary integration sites for the complex regulation of growth cone motility, cell division, and actin dynamics in neuronal growth [4], [46]. Over expression of dominant active forms of Rho initiate axon outgrowth and control of growth cone filopodial dynamics and promote axonal regeneration [4], [47]. Rho proteins, like other members of the Ras superfamily, have been shown to control the actin cytoskeleton by the active cycle changes between active GTP-bound and inactive GDP-bound states. p75NTR interacts with Rho-GDI which is stimulated by MAG, initiating the activation of RhoA to inhibit the growth [48], [49]. We found that RhoA-GTPase is immediately activated in response to proBDNF, which was dependent on p75NTR. It is likely that the binding of proBDNF to p75NTR directly stimulates the interaction with Rho-GDI and promotes the generation of RhoA-GTPase which causes the subsequent collapse growth cones and filopodia.

The activation of RhoA and its downstream effector ROCK leads to growth cone collapse and neurite growth arrest, a mechanism that has been extensively studied in experiments on the myelin-associated inhibitors MAG, Nogo, and OMgp [50], [51]. To directly link the proBDNF-induced activation of RhoA and ROCK with inhibition of neurite outgrowth, pharmacological approaches were used to prevent the activation to abrogate the deleterious effects of the proBDNF. The reversal of inhibition with C3-transferase was less than that of Y27632, and this may be attributable to relatively lower cell permeability of C3-transferase [52]. Our studies show that the inhibition induced by proBDNF is via engagement of activation of RhoA and its downstream effector ROCK.

In the present study, using p75NTR^−/−^ neurons and mice we could show that p75NTR is the proBDNF downstream signal molecule which is essential for the neurite collapse. We found that proBDNF neurite collapsing effect *in vitro* and *in vivo* are lost in p75NTR^−/−^ neurons and in p75NTR^−/−^ mice. Furthermore, we found that the RhoA activation by proBDNF was also abolished in p75NTR^−/−^ neurons. These data indicate that p75NTR is responsible for the action of proBDNF. Our data raise a question of how to reconcile the contradictory roles of p75NTR in neurite growth/collapse. Yamashita *et al.* showed that mature neurotrophins inhibit RhoA in the absence of Trk receptors, promoting neurite growth [48]. Recently, Yamashita’s group discovered another pathway demonstrating that p75NTR may activate paired immunoglobulin-like receptor B (PIR-B) which recruits Src homology 2-containing protein tyrosine phosphatise (SHP)-1 and SHP-2, that dephosphorylates Trk receptors and reduces neurite growth [53]. The activation by mature neurotrophins of p75NTR in the presence or absence of Trk receptors would promote neurite growth via inactivation of RhoA and/or activation of the phosphatidylinositide 3 kinase (PI3K) and the mitogen-activated protein kinase (MAPK) pathway [48], [54], [55], [56]. On the other hand, the activation of p75NTR by proneurotrophins and/or myelin inhibitory factors in the absence of Trk or low levels Trk receptors in neurons would activate RhoA and suppress the signal transduction of Trk/MAPK, leading to the neurite collapse and degeneration. Thus, activation or inactivation of RhoA signaling pathway via the p75NTR is ligand- and coreceptor-dependent. The double-faced actions of p75NTR on neurite growth explains why no enhanced nerve regeneration is seen in p75NTR^−/−^ mice after spinal cord injury [57].

The function of p75NTR in neurite growth does not only depend on neuronal expression of p75NTR, but also on the glial expression of p75NTR [58]. This interrelationship between different cell types expressing p75NTR is even more complicated in the peripheral nervous system. For example, macrophages express p75NTR and have been reported to affect peripheral nerve regeneration [59], p75NTR is highly expressed by peripheral neurons and also Schwann cells after Wallerian degeneration. We and others showed that Schwann cell p75NTR positively regulates nerve regeneration. The depletion of p75NTR causes retardation of peripheral nerve regeneration after nerve injury [57], [60], [61] and reduces the nerve elongation during development [32], [62].

In summary, proBDNF is a powerful neurite outgrowth inhibitor which activates RhoA via p75NTR. ProBDNF also decreases filopodial length and number of growth cones and triggers a polarized distribution of activated RhoA in the tips of growth cones. Neutralization of endogenous proBDNF enhances the innervation. Endogenous proBDNF may play an opposing role to that of mature BDNF regulating neurite growth.

## Materials and Methods

### Animals and Reagents

All experimental procedures were under the guidelines of the National Health and Medical Research Council of Australia and approved by the Animal Welfare Committee of Flinders University. Adult 8–10 weeks old Sprague-Dawley (SD) rats (*n* = 12), neonatal 129 sv wild-type (p75NTR^+/+^, *n* = 24), postnatal day 5 (P5, p75NTR^+/+^, *n* = 15), p75NTR deficient mice (p75NTR^−/−^, *n* = 12) were used.

Recombinant proBDNF with a RR-AA mutation of the cleavage site and the neutralizing proBDNF antiserum were produced in our lab and their biological activities have been characterized [63]. The antisera directed against proBDNF for immunohistochemistry have been characterised [27]. The neutralizing antiserum specifically recognize proBDNF but not mature BDNF and other neurotrophins [11], [12], [25], [26], [63]. Culture media were obtained from Invitrogen and other reagents were obtained from Sigma (St. Louis, MO) unless specified otherwise.

### Primary Neuron Culture

Lumbar dorsal root ganglia (DRG) were dissected from SD rats (∼250 g, *n* = 6) and cut into eight pieces, digested at Collagenase II Ca^2+^ free Hank’s solution as described previously [64]. The tissues were dissociated using a fire-polished Pasteur pipette, the cell suspension was layered on top of a 15% fatty acid-free albumin bovine serum (BSA, Sigma) solution. After a 400 *g* centrifugation for 20 min the pellet was resuspended in Neurobasal medium (Invitrogen) containing 0.25% foetal calf serum (FCS, Invitrogen), penicillin–streptomycin (100 U), L-glutamine (2 mM) and B27 (2%) serum-free supplement (Invitrogen). The number of viable cells was determined by trypan blue (Sigma) exclusion and 2×10^4^ DRG cells were plated onto poly-D-lysine-coated cell culture dishes and cultured at 37°C in 95% O_2_ ? 5% CO_2_ incubator. Cultured DRG neurons were treated with proBDNF (0, 3, 10, 30, 100 ng/ml), anti-proBDNF (10 µg/ml), BDNF (50 ng/ml), normal sheep IgG (IgG, 10 µg/ml) or recombinant rat MAG-Fc chimera (0, 3, 10, 30 µg/ml, R&D Systems, USA).

For primary cortical neuron cultures, p75NTR^+/+^ (*n* = 12), p75NTR^−/−^ (*n* = 6) mice postnatal day 1 (P1) were killed, neurons were purified as described previously [11], [65]. Briefly, cortices were collected, meninges removed and tissue sliced into 0.5 mm pieces. Tissue blocks were incubated in 1% trypsin ? EDTA (Invitrogen, Australia) with 0.1% DNaseI (Worthington, NJ, USA) for 8 min at 37°C with shaking every 5 min. Digestion was stopped by adding 15% FCS. After a 400 *g* centrifugation for 10 min the pellet was resuspended in 1 ml Neurobasal medium supplemented with B27 (2%), N2 (1%), penicillin–streptomycin (100 U), L-glutamine (2 mM) and FCS (2%). 1×10^6^ cortical cells were plated onto poly-D-lysine-coated cell culture dishes, after 24 h cultured at 37°C in 95% O_2_ ? 5% CO_2_ incubator, neurons were incubated with BSA as a control, proBDNF or MAG. Neurite length was measured after 2 h treatment.

### Time-lapse Video Microscopy

Phase-contrast images from cultured DRG neurons were collected using an inverted microscope (BioStation, Nikon, Japan). Digital images were captured every 30 sec for 30 min, collected individually and analyzed by Advanced Research software for the determination of the neurite extension length. The extension rate was defined as the difference between the length at time point 0 and the time examined and divided by the time period (seconds).

### Western Blot of ProBDNF, p75NTR and Sortilin in Cortex or DRG

The cultured neuron protein concentration for Western Blot was assayed using a BCA™ Protein Assay Kit (Pierce, Rockford, IL, USA). 40 µg of homogenate proteins were denatured by boiling for 5 min and separated by sodium dodecyl sulphate-polyacrylamide gel electrophoresis (SDS-PAGE). The nitrocellulose membrane (0.45 µm pore size, GE Healthcare Life Science) was probed with primary antibodies directed to rabbit anti-p75NTR (1∶1000, G3231, Promega, USA), rabbit anti-sortilin (1∶1000, Osenses, Australia) and a monoclonal antibody to β-actin (1∶1000, Sigma) as the internal control overnight, respectively, followed by incubation with the horseradish peroxidase (HRP)-linked secondary antibodies (Jackson Laboratories) at 1∶2000 for 1 h at room temperature. Membranes were developed by using the enhanced chemiluminescence (ECL) system (Amersham Biosciences).

### Indirect Immunofluorescence and Neurite Outgrowth Assay

For outgrowth assays, primary cultured neurons were fixed with 4% paraformaldehyde (PFA), then incubated overnight with 1∶1000 rabbit anti-MAP2 polyclonal antibody (Osenses, Australia) followed by incubation with Alexa Fluor 488 anti-rabbit secondary antibody (Jackson Laboratories, USA). Images were acquired from randomly selected fields (*n* = 8∼12) under Fluorescence Microscope (Olympus BX50). The length of the longest neurite of 80∼120 neurons per condition was determined using ImageJ software [66]. Each experimental condition was done in duplicate wells, and at least three independent experiments were conducted to acquire the final results.

### Activation Assay of RhoA and Cdc42

The proBDNF-dependent activation of RhoA and Cdc42 was assessed using a pull-down assay kit according to manufacturer instructions (NewEast Biosciences, USA). Due to the limited number of DRG neurons we used cortical neurons in this assay. 10^7^ cortical cells from p75NTR^+/+^ and p75NTR^−/−^ mice were cultured and treated with 30 ng/ml proBDNF for 0, 5, 10, 20 min, and subsequently homogenized and lysed with RIPA buffer containing protease inhibitors (Roche Applied Science). One aliquot was used for immunoblot (IB) analysis, the second aliquot was incubated with GTP-RhoA or GTP-Cdc42 antibody, bound with protein A/G agarose beads for 1 h at 4 °C, precipitated and subjected to SDS-PAGE, detected by anti-RhoA or anti-Cdc42 antibodies. GTP-activated levels were normalized to the corresponding total RhoA levels using densitometric analysis using ImageJ software. The data were expressed as a percentage of the signals obtained at 5, 10 and 20 min in comparison with the time point 0.

### Immunohistochemical Detection of Activated RhoA and Cdc42

Primary cultured rat DRG neurons (*n* = 6) were exposed to proBDNF for 0, 5, 10, 20 min, fixed with 4% PFA and incubated with antisera directed against GTP-RhoA or GTP-Cdc42 (NewEast Biosciences, USA) respectively, followed by incubation with Alexa Fluro 594 anti-mouse secondary antibody (Jackson Laboratories, USA). The presence of GTP-activated RhoA or Cdc42 in growth cones was quantified by measuring signal intensity in each growth cone using ImageJ. For F-actin staining, primary cultured DRG neurons were fixed with 0.25% glutaraldehyde, quenched with 1 mg/ml sodium borohydride for 15 min, followed by incubation rhodamine-conjugated phalloidin (Molecular Probes, USA). The staining of growth cones were captured using a confocal microscope (Leica SP5, Germany).

### Neurite Outgrowth Assay Detects Activation of RhoA and ROCK Pathway

The chemical approach included treatment of cultured DRG neurons with the RhoA inhibitor, C3-transferase (25 µg/ml; Cytoskeleton), and/or the ROCK inhibitor, Y27632 (10 µM; Calbiochem), before adding proBDNF (30 ng/ml). 2×10^4^ cultured DRG neurons were treated after 24 h later, neurite length was measured as above mentioned. Cell lysates were assayed for activated RhoA and for phosphorylated CRMP-2 (rabbit polyclonal anti phosphorylated CRMP-2 affinity purified antibody was a gift from Dr S. Petratos, Monash University, Clayton, Australia).

### *In vivo* Delivery of Exogenous ProBDNF and its Neutralizing Antiserum into the Footpad of Pups

10 µl of proBDNF (1 µg), anti-proBDNF (5 µg) or IgG (5 µg) were injected into the footpad of P5 p75NTR^+/+^ and p75NTR^−/−^ mice (*n* = 5/treatment) on three consecutive days. On day ten, the pups were anesthetized and immobilized perfused through the heart with 30 ml saline followed by 50 ml Zamboni’s fixative as described previously [11]. After perfusion, the injected ventral planta was dissected and post-fixed overnight in the same fixative. The next day, the planta was cryopreserved in 0.1 M phosphate-buffered saline +18% sucrose. Sagittal cryostat sections (30 µm thickness) were cut into five series using a freezing microtome (Leica, Germany) and mounted on gelatine-coated glass slides as described previously [11]. The series in every fifth mid-sagittal sections were used for nerve fiber density quantification. These sections were blocked using 5% donkey serum for 1 h at room temperature then incubated with anti-neurofilament antibody (1∶1000, RT97, Abcam, UK) overnight at 4°C, followed by incubation with Alexa Fluor 594 anti-mouse secondary antibody. Images were taken from 8 sections per animal under Fluorescence Microscope (Olympus BX50). The nerve fiber density was determined by determing the area occupied by nerve fibers at 150 µm intervals using ImageJ program. Five animals per treatment were used in this study. The data were expressed as a normalized nerve fiber density using normal sheep IgG group as 1.

### Statistical Analysis

The *in vivo* experiments of footpad injection were carried out in a double blinded manner. The data are expressed as mean ± standard error of the mean (SEM). Experiments with intra or inter group were analyzed by using Student’s *t* test or one way ANOVA with Least-significant Difference (LSD) Post Hoc Test. *p* values<0.05 were considered statistically significant. All statistical analyses of the experimental data were performed using 13.0 SPSS.

## Supporting Information

Figure S1 ProBDNF treatment decreases neurite length on cultured DRG neurons.
(AVI)

Figure S2 BDNF treatment increases neurite length on cultured DRG neurons.
(AVI)

## References

[1] Bradke F, Dotti CG (2000). Differentiated neurons retain the capacity to generate axons from dendrites.. Curr Biol.

[2] Conde C, Caceres A (2009). Microtubule assembly, organization and dynamics in axons and dendrites.. Nat Rev Neurosci.

[3] Cohen-Cory S, Fraser SE (1995). Effects of brain-derived neurotrophic factor on optic axon branching and remodelling in vivo.. Nature.

[4] Gehler S, Gallo G, Veien E, Letourneau PC (2004). p75 neurotrophin receptor signaling regulates growth cone filopodial dynamics through modulating RhoA activity.. J Neurosci.

[5] Lu B, Pang PT, Woo NH (2005). The yin and yang of neurotrophin action.. Nat Rev Neurosci.

[6] Kenchappa RS, Zampieri N, Chao MV, Barker PA, Teng HK (2006). Ligand-dependent cleavage of the P75 neurotrophin receptor is necessary for NRIF nuclear translocation and apoptosis in sympathetic neurons.. Neuron.

[7] Yano H, Torkin R, Martin LA, Chao MV, Teng KK (2009). Proneurotrophin-3 is a neuronal apoptotic ligand: evidence for retrograde-directed cell killing.. J Neurosci.

[8] Yang F, Je HS, Ji Y, Nagappan G, Hempstead B (2009). Pro-BDNF-induced synaptic depression and retraction at developing neuromuscular synapses.. J Cell Biol.

[9] Woo NH, Teng HK, Siao CJ, Chiaruttini C, Pang PT (2005). Activation of p75NTR by proBDNF facilitates hippocampal long-term depression.. Nat Neurosci.

[10] Koshimizu H, Kiyosue K, Hara T, Hazama S, Suzuki S (2009). Multiple functions of precursor BDNF to CNS neurons: negative regulation of neurite growth, spine formation and cell survival.. Mol Brain.

[11] Xu ZQ, Sun Y, Li HY, Lim Y, Zhong JH (2011). Endogenous proBDNF is a negative regulator of migration of cerebellar granule cells in neonatal mice.. Eur J Neurosci.

[12] Wong I, Liao H, Bai X, Zaknic A, Zhong J (2010). ProBDNF inhibits infiltration of ED1+macrophages after spinal cord injury.. Brain Behav Immun.

[13] Wang KC, Kim JA, Sivasankaran R, Segal R, He Z (2002). P75 interacts with the Nogo receptor as a co-receptor for Nogo, MAG and OMgp.. Nature.

[14] Woolf CJ, Bloechlinger S (2002). Neuroscience. It takes more than two to Nogo.. Science.

[15] Tang XQ, Tanelian DL, Smith GM (2004). Semaphorin3A inhibits nerve growth factor-induced sprouting of nociceptive afferents in adult rat spinal cord.. J Neurosci.

[16] Benson MD, Romero MI, Lush ME, Lu QR, Henkemeyer M (2005). Ephrin-B3 is a myelin-based inhibitor of neurite outgrowth.. Proc Natl Acad Sci U S A.

[17] Liu X, Hashimoto M, Horii H, Yamaguchi A, Naito K (2009). Repulsive guidance molecule b inhibits neurite growth and is increased after spinal cord injury.. Biochem Biophys Res Commun.

[18] Naska S, Lin DC, Miller FD, Kaplan DR (2010). p75NTR is an obligate signaling receptor required for cues that cause sympathetic neuron growth cone collapse.. Mol Cell Neurosci.

[19] Teng HK, Teng KK, Lee R, Wright S, Tevar S (2005). ProBDNF induces neuronal apoptosis via activation of a receptor complex of p75NTR and sortilin.. J Neurosci.

[20] Yamashita T, Tohyama M (2003). The p75 receptor acts as a displacement factor that releases Rho from Rho-GDI.. Nat Neurosci.

[21] Gallo G, Letourneau PC (2000). Neurotrophins and the dynamic regulation of the neuronal cytoskeleton.. J Neurobiol.

[22] Marler KJ, Poopalasundaram S, Broom ER, Wentzel C, Drescher U (2010). Pro-neurotrophins secreted from retinal ganglion cell axons are necessary for ephrinA-p75NTR-mediated axon guidance.. Neural Dev.

[23] Nagappan G, Zaitsev E, Senatorov VV, Yang J, Hempstead BL (2009). Control of extracellular cleavage of ProBDNF by high frequency neuronal activity.. Proc Natl Acad Sci U S A.

[24] Yang J, Siao CJ, Nagappan G, Marinic T, Jing D (2009). Neuronal release of proBDNF.. Nat Neurosci.

[25] Wang H, Wu LL, Song XY, Luo XG, Zhong JH (2006). Axonal transport of BDNF precursor in primary sensory neurons.. Eur J Neurosci.

[26] Wu LL, Fan Y, Li S, Li XJ, Zhou XF (2010). Huntingtin-associated protein-1 interacts with pro-brain-derived neurotrophic factor and mediates its transport and release.. J Biol Chem.

[27] Zhou XF, Song XY, Zhong JH, Barati S, Zhou FH (2004). Distribution and localization of pro-brain-derived neurotrophic factor-like immunoreactivity in the peripheral and central nervous system of the adult rat.. J Neurochem.

[28] Matsumoto T, Rauskolb S, Polack M, Klose J, Kolbeck R (2008). Biosynthesis and processing of endogenous BDNF: CNS neurons store and secrete BDNF, not pro-BDNF.. Nat Neurosci.

[29] Lee R, Kermani P, Teng KK, Hempstead BL (2001). Regulation of cell survival by secreted proneurotrophins.. Science.

[30] Fahnestock M, Yu G, Michalski B, Mathew S, Colquhoun A (2004). The nerve growth factor precursor proNGF exhibits neurotrophic activity but is less active than mature nerve growth factor.. J Neurochem.

[31] Wong ST, Henley JR, Kanning KC, Huang KH, Bothwell M (2002). A p75(NTR) and Nogo receptor complex mediates repulsive signaling by myelin-associated glycoprotein.. Nat Neurosci.

[32] Bentley CA, Lee KF (2000). p75 is important for axon growth and schwann cell migration during development.. J Neurosci.

[33] Walsh GS, Krol KM, Crutcher KA, Kawaja MD (1999). Enhanced neurotrophin-induced axon growth in myelinated portions of the CNS in mice lacking the p75 neurotrophin receptor.. J Neurosci.

[34] Singh KK, Park KJ, Hong EJ, Kramer BM, Greenberg ME (2008). Developmental axon pruning mediated by BDNF-p75NTR-dependent axon degeneration.. Nat Neurosci.

[35] Ahmed Z, Mazibrada G, Seabright RJ, Dent RG, Berry M (2006). TACE-induced cleavage of NgR and p75NTR in dorsal root ganglion cultures disinhibits outgrowth and promotes branching of neurites in the presence of inhibitory CNS myelin.. FASEB J.

[36] Jansen P, Giehl K, Nyengaard JR, Teng K, Lioubinski O (2007). Roles for the pro-neurotrophin receptor sortilin in neuronal development, aging and brain injury.. Nat Neurosci.

[37] McKerracher L, David S, Jackson DL, Kottis V, Dunn RJ (1994). Identification of myelin-associated glycoprotein as a major myelin-derived inhibitor of neurite growth.. Neuron.

[38] Cai D, Shen Y, De Bellard M, Tang S, Filbin MT (1999). Prior exposure to neurotrophins blocks inhibition of axonal regeneration by MAG and myelin via a cAMP-dependent mechanism.. Neuron.

[39] Lee JK, Zheng B (2011). Role of myelin-associated inhibitors in axonal repair after spinal cord injury..

[40] Lee JK, Kim JE, Sivula M, Strittmatter SM (2004). Nogo receptor antagonism promotes stroke recovery by enhancing axonal plasticity.. J Neurosci.

[41] Zheng B, Ho C, Li S, Keirstead H, Steward O (2003). Lack of enhanced spinal regeneration in Nogo-deficient mice.. Neuron.

[42] Lee JK, Chow R, Xie F, Chow SY, Tolentino KE (2010). Combined genetic attenuation of myelin and semaphorin-mediated growth inhibition is insufficient to promote serotonergic axon regeneration.. J Neurosci.

[43] Lee JK, Geoffroy CG, Chan AF, Tolentino KE, Crawford MJ (2010). Assessing spinal axon regeneration and sprouting in Nogo-, MAG-, and OMgp-deficient mice.. Neuron.

[44] Kater SB, Rehder V (1995). The sensory-motor role of growth cone filopodia.. Curr Opin Neurobiol.

[45] Wang GX, Poo MM (2005). Requirement of TRPC channels in netrin-1-induced chemotropic turning of nerve growth cones.. Nature.

[46] Kuhn TB, Meberg PJ, Brown MD, Bernstein BW, Minamide LS (2000). Regulating actin dynamics in neuronal growth cones by ADF/cofilin and rho family GTPases.. J Neurobiol.

[47] Bito H, Furuyashiki T, Ishihara H, Shibasaki Y, Ohashi K (2000). A critical role for a Rho-associated kinase, p160ROCK, in determining axon outgrowth in mammalian CNS neurons.. Neuron.

[48] Yamashita T, Tucker KL, Barde YA (1999). Neurotrophin binding to the p75 receptor modulates Rho activity and axonal outgrowth.. Neuron.

[49] Park KJ, Grosso CA, Aubert I, Kaplan DR, Miller FD (2010). p75NTR-dependent, myelin-mediated axonal degeneration regulates neural connectivity in the adult brain.. Nat Neurosci.

[50] Grados-Munro EM, Fournier AE (2003). Myelin-associated inhibitors of axon regeneration.. J Neurosci Res.

[51] McGee AW, Strittmatter SM (2003). The Nogo-66 receptor: focusing myelin inhibition of axon regeneration.. Trends Neurosci.

[52] Zhang G, Lehmann HC, Manoharan S, Hashmi M, Shim S (2011). Anti-ganglioside antibody-mediated activation of RhoA induces inhibition of neurite outgrowth.. J Neurosci.

[53] Fujita Y, Endo S, Takai T, Yamashita T (2011). Myelin suppresses axon regeneration by PIR-B/SHP-mediated inhibition of Trk activity.. EMBO J.

[54] Kaplan DR, Miller FD (2000). Neurotrophin signal transduction in the nervous system.. Curr Opin Neurobiol.

[55] Nguyen N, Lee SB, Lee YS, Lee KH, Ahn JY (2009). Neuroprotection by NGF and BDNF against neurotoxin-exerted apoptotic death in neural stem cells are mediated through Trk receptors, activating PI3-kinase and MAPK pathways.. Neurochem Res.

[56] Huang EJ, Reichardt LF (2003). Trk receptors: roles in neuronal signal transduction.. Annu Rev Biochem.

[57] Song XY, Zhong JH, Wang X, Zhou XF (2004). Suppression of p75NTR does not promote regeneration of injured spinal cord in mice.. J Neurosci.

[58] Zhou XF, Li HY (2007). Roles of glial p75NTR in axonal regeneration.. J Neurosci Res.

[59] Fry EJ, Ho C, David S (2007). A role for Nogo receptor in macrophage clearance from injured peripheral nerve.. Neuron.

[60] Tomita K, Kubo T, Matsuda K, Fujiwara T, Yano K (2007). The neurotrophin receptor p75NTR in Schwann cells is implicated in remyelination and motor recovery after peripheral nerve injury.. Glia.

[61] Song XY, Zhang FH, Zhou FH, Zhong J, Zhou XF (2009). Deletion of p75NTR impairs regeneration of peripheral nerves in mice.. Life Sci.

[62] Lee KF, Li E, Huber LJ, Landis SC, Sharpe AH (1992). Targeted mutation of the gene encoding the low affinity NGF receptor p75 leads to deficits in the peripheral sensory nervous system.. Cell.

[63] Fan YJ, Wu LL, Li HY, Wang YJ, Zhou XF (2008). Differential effects of pro-BDNF on sensory neurons after sciatic nerve transection in neonatal rats.. Eur J Neurosci.

[64] Bastian I, Tam Tam S, Zhou XF, Kazenwadel J, Van der Hoek M (2011). Differential expression of microRNA-1 in dorsal root ganglion neurons.. Histochem Cell Biol.

[65] Wang YJ, Valadares D, Sun Y, Wang X, Zhong JH (2010). Effects of proNGF on neuronal viability, neurite growth and amyloid-beta metabolism.. Neurotox Res.

[66] Wu D, Huang W, Richardson PM, Priestley JV, Liu M (2008). TRPC4 in rat dorsal root ganglion neurons is increased after nerve injury and is necessary for neurite outgrowth.. J Biol Chem.

